# Bridging the visual-to-physical gap: physically aligned representations for fall risk analysis

**DOI:** 10.3389/fmed.2026.1837781

**Published:** 2026-05-05

**Authors:** Xianqi Zhang, Xingtao Wang, Xiaopeng Fan

**Affiliations:** 1 Faculty of Computing, Harbin Institute of Technology, Harbin, China; 2 Suzhou Research Institute, Harbin Institute of Technology, Suzhou, China; 3 Peng Cheng Laboratory, Shenzhen, China

**Keywords:** contrastive learning, deep learning, embedding geometry, fall risk analysis, representation learning

## Abstract

**Introduction:**

Vision-based fall analysis has advanced rapidly, yet a key bottleneck remains: visually similar motions can correspond to markedly different physical outcomes, as subtle differences in contact mechanics and protective responses are difficult to infer from appearance alone. Existing approaches typically rely on supervised injury prediction, which requires reliable clinical labels. In practice, such labels are difficult to obtain due to ambiguity in video evidence (e.g., occlusion and viewpoint limitations) and the rarity and ethical constraints of real injury events, leading to noisy supervision.

**Methods:**

We propose PHARL (PHysics-aware Alignment Representation Learning), a framework that learns physically meaningful fall representations without requiring clinical outcome labels. PHARL introduces two complementary regularization mechanisms: (1) trajectory-level temporal consistency to stabilize motion representations, and (2) physics-aware alignment, where simulation-derived multi-class contact outcomes are used to structure the embedding space. By associating video windows with temporally aligned simulation descriptors, the model captures local impact-relevant dynamics while maintaining a purely feed-forward inference process. Notably, physics information is only used during training as a structural regularizer and does not define an explicit outcome predictor.

**Results:**

Experiments on four public datasets demonstrate that PHARL consistently improves risk-aligned representation quality compared to visual-only baselines, while maintaining strong performance on fall detection tasks.

**Discussion:**

Beyond quantitative improvements, PHARL exhibits an emergent ordinal structure in the learned representation space, where an interpretable severity ordering (Head > Trunk > Supported) arises without explicit ordinal supervision. This suggests that physics-aware regularization can induce meaningful structural priors in representation learning, offering a promising direction for label-efficient fall analysis.

## 1 Introduction

Falls are complex events involving fast posture transitions and contact interactions. Although computer vision has made strong progress in binary fall detection (1, 2), a central challenge remains: *kinematic-to-physical ambiguity*. In practice, trajectories that look similar can still lead to very different outcomes, depending on subtle protective responses and contact topology (e.g., arm-braced landing vs. direct head impact) (3, 4). For purely data-driven models, separating these cases is difficult despite their markedly different physical consequences.

Recent developments in this field have mainly focused on stronger spatiotemporal modeling and large-scale representation learning. Deep learning-based fall detection methods have been developed for a long time (5, 6). In parallel, contrastive and masked pretraining paradigms have significantly improved motion representation quality in action understanding (7–11). However, most of these advances still optimize visual or temporal discrimination, and therefore provide limited supervision for distinguishing physically distinct outcomes within the same “fall” category.

Beyond binary detection, current research has started to address fall risk and post-fall consequence analysis, including gait-based risk assessment (12), privacy-preserving anomaly modeling (13, 14), and sensor-fusion or skeleton-centric pipelines for robustness in real environments (15). Nevertheless, most practical systems are still optimized for event detection rather than physically grounded outcome characterization. As a result, controlled low-impact falls and high-impact falls may still be represented too similarly when supervision is limited to coarse labels.

Traditional outcome-oriented strategies attempt to predict injury severity directly from labeled data. Although intuitive, this route depends on reliable injury annotations, which are difficult to acquire in realistic settings. Visual evidence is often ambiguous because of occlusions and viewpoint limitations, and genuine injury outcomes are rare and cannot be safely staged. As a result, direct supervision is inherently noisy. Motivated by this limitation, we shift the objective from outcome prediction to representation shaping: the embedding should encode physics-consistent contact structure so that downstream analysis can separate outcome-relevant differences without dense clinical labels.

To this end, we introduce PHARL (PHysics-aware Alignment Representation Learning), which uses physics simulation as structural supervision rather than as a direct prediction target. Concretely, estimated human motion is retargeted to a high-fidelity humanoid model, and short-horizon simulation produces coarse contact outcomes. We use a humanoid simulation proxy because PHARL only requires coarse, relative contact-structure signals to regularize representation geometry, rather than subject-specific injury biomechanics. Given that ordinary RGB datasets do not provide reliable personalized biomechanical parameters, a unified humanoid model offers a practical balance of physical plausibility, simulation stability, and scalability across datasets. We therefore treat simulation outputs as structural weak supervision, not as patient-level biomechanical predictions. PHARL is best understood as a redesign of contrastive relations: physics-derived supervision modifies pair construction and pair weighting within a standard contrastive objective, rather than introducing a separate physics-prediction task. These outcomes define cross-trajectory equivalence relations that regularize latent-space geometry through multi-class physics alignment. Consequently, the learned representation exhibits a consistent emergent ordinal trend with respect to contact outcome.

This design is intentionally non-predictive: PHARL does not output clinical diagnosis, injury probability, or deployable severity scores. Here, “non-predictive” refers to the training objective, rather than to the absence of *post-hoc* probing analyses. Instead, physics-derived outcomes—obtained without manual injury annotation—are only used to build contrastive relations and neighborhood constraints in representation space. Compared with purely motion-driven objectives, this formulation better separates physically distinct cases (e.g., successful upper-limb protection vs. direct head-ground contact), while preserving efficient feed-forward inference because simulation is only required during training.

The main contributions of this paper are summarized as follows:

1. We reformulate fall analysis as a weakly supervised representation learning problem, where physics-derived contact outcomes provide structural supervision without training an explicit outcome predictor.
2. We propose a physics-consistency objective that regularizes embedding geometry through denominator masking (false-negative removal) and auxiliary physics-aligned positives, using physics-derived equivalence instead of manual outcome labels.
3. We establish a rank-prioritized evaluation protocol to assess both utility and interpretability, including emergent ordinal structure diagnostics (Spearman ρ, POA), contact localization (Binary Contact AP/AUC), fall-detection sanity checks (AUC), and geometric diagnostics (PCR, Kendall τ).

The remainder of this paper is organized as follows. Section 2 reviews related work. Section 3 presents the PHARL framework, including motion-level temporal consistency and physics-level outcome consistency. Section 4 describes the experimental setup and results. Section 5 discusses implications and limitations, and Section 6 concludes the paper.

## 2 Related work

### 2.1 Fall detection and outcome analysis

Vision-based fall analysis has evolved from handcrafted descriptors to deep spatiotemporal modeling. Early studies formulated fall detection as action classification with manually designed motion features (16). With the growth of public datasets such as Le2i, URFD, MCFD, and CAUCAFall (17–20), deep-learning based methods became the dominant paradigm for capturing scene context and temporal cues under more realistic conditions.

Existing studies show that deep learning-based pipelines and multimodal fusion strategies achieve improved performance, particularly in complex daily-activity scenarios (1, 2, 5, 6). Skeleton-centered approaches further improve robustness by reducing appearance bias and focusing on motion structure (15). Transformer-based architectures then extend this direction by modeling long-range dependencies and reducing false alarms caused by visually similar non-fall activities.

Despite these advances, most systems are still optimized for binary event recognition (fall vs. non-fall), where physically different post-fall outcomes may share the same supervision signal. In practical safety monitoring, this leaves a gap between detection capability and outcome-aware interpretation, particularly when only weak labels are available.

A parallel line of research targets fall risk estimation and prevention rather than post-fall representation. Representative efforts include gait-informed risk assessment (21, 22), anomaly-based monitoring under privacy constraints (13, 14). These studies are crucial for prevention, but they generally do not learn contact-aware latent structures for post-fall motion outcome analysis.

### 2.2 Contrastive representation learning for human motion

Self-supervised contrastive learning has become a core strategy for representation learning without dense annotation (23–25). In video and motion understanding, temporal consistency and instance discrimination objectives improve transferability across downstream tasks (7, 8). Recent methods extend these ideas with harder negative mining and fine-grained positive construction to improve intra-class discrimination (26, 27).

For skeleton and human-motion domains, large-scale pretraining methods such as MotionBERT (9) and SkeletonMAE (10) report strong gains in representation quality. Related frameworks including multi-skeleton and mask-based contrastive variants further demonstrate that objective design substantially influences learned motion semantics (28). Benchmark studies and surveys confirm that the field is moving toward broader pretraining and stronger invariance learning (11, 29, 30).

However, the invariances encouraged by standard contrastive objectives are not always aligned with fall-outcome understanding. Moreover, temporal proximity alone can create positive pairs that are visually similar but outcome-inconsistent, which limits direct applicability to contact-sensitive fall representation.

### 2.3 Physics-informed learning for human dynamics

Physics-informed learning introduces domain priors to improve plausibility and consistency in human motion modeling. Broadly, existing methods rely on soft constraints (e.g., penetration/contact penalties) or hard constraints through differentiable simulation and dynamics optimization (31–33). In synthesis and forecasting tasks, physics-guided frameworks such as PhysDiff (34) and PIMNet (35) show clear improvements in realism and physical feasibility.

Recent studies also explore integrating world models, physics simulators, and embodied priors into perception pipelines (36–38). These methods demonstrate that simulation signals can regularize representations beyond pure appearance cues, especially when real annotations are sparse.

Compared with prior physics-informed work, PHARL does not use physics to generate trajectories at deployment time, nor to train an end-to-end outcome classifier. Instead, simulation is used during training to construct weak structural supervision in embedding space. This design preserves inference efficiency while injecting contact-relevant physical structure. Unlike structural-prior approaches that regularize single-trajectory dynamics or reconstruction targets, PHARL uses simulation to redefine inter-sample relations in contrastive space through cross-trajectory equivalence and false-negative masking.

In summary, existing fall-detection literature provides strong event-level discrimination, contrastive learning offers scalable representation learning, and physics-informed methods contribute physical plausibility. Yet their intersection remains underdeveloped for outcome-aware fall representation under scarce clinical labels. PHARL targets this intersection by combining contrastive learning with simulation-derived outcome equivalence, aiming to improve contact-aware organization without requiring injury labels or simulation at inference time.

## 3 Method

### 3.1 Problem formulation

We study outcome-aware representation learning for fall videos without injury annotations. Let $X=\left\{x_{1},x_{2},\ldots ,x_{N}\right\}$ denote a dataset of RGB clips depicting falls or daily activities. Each clip *x*_*i*_ has *T* frames, but no clinical injury label $y_{i}^{\text{injury}}$ is available. 3D pose/SMPL information is used only offline to run simulation and construct physics/temporal relations; it is never used as encoder input and is unavailable at inference time.

Our goal is to learn an encoder $f:x_{i}\mapsto z_{i}\in {\mathbb{R}}^{d}$ that maps motion observations to embeddings satisfying three properties:

1. Motion coherence: Temporally adjacent clips from the same sequence map to nearby embeddings, ensuring temporal continuity.
2. Outcome consistency: Clips leading to similar physics-derived contact outcomes map to nearby embeddings, even if their visual trajectories differ.
3. Non-predictive semantics: The representation encodes outcome-aware structure without training an outcome predictor or enforcing ordinal ranking; any ordering observed in the embedding space is evaluated *post-hoc* for analysis only.^1^

PHARL is formulated at the temporal-window level rather than the full-video level. We adopt this setting because a single fall trajectory can contain multiple physical phases (pre-impact, trunk impact, head impact), which are difficult to represent with one video-level label.

These properties must emerge without direct supervision on injury outcomes. In practice, training uses only trajectory identity and physics-derived weak labels; no clinical target is optimized. Consequently, evaluation can only assess representation structure—such as contact-aware separability and physics-consistent geometry—rather than predictive performance on injury classification.

### 3.2 Overview of PHARL framework

Mechanistically, temporal consistency stabilizes local motion neighborhoods within individual trajectories, whereas physics alignment imposes cross-trajectory structure among windows that share similar contact outcomes. Their interaction reduces kinematic aliasing and promotes an emergent ordinal organization in the embedding space.

**Figure 1:**
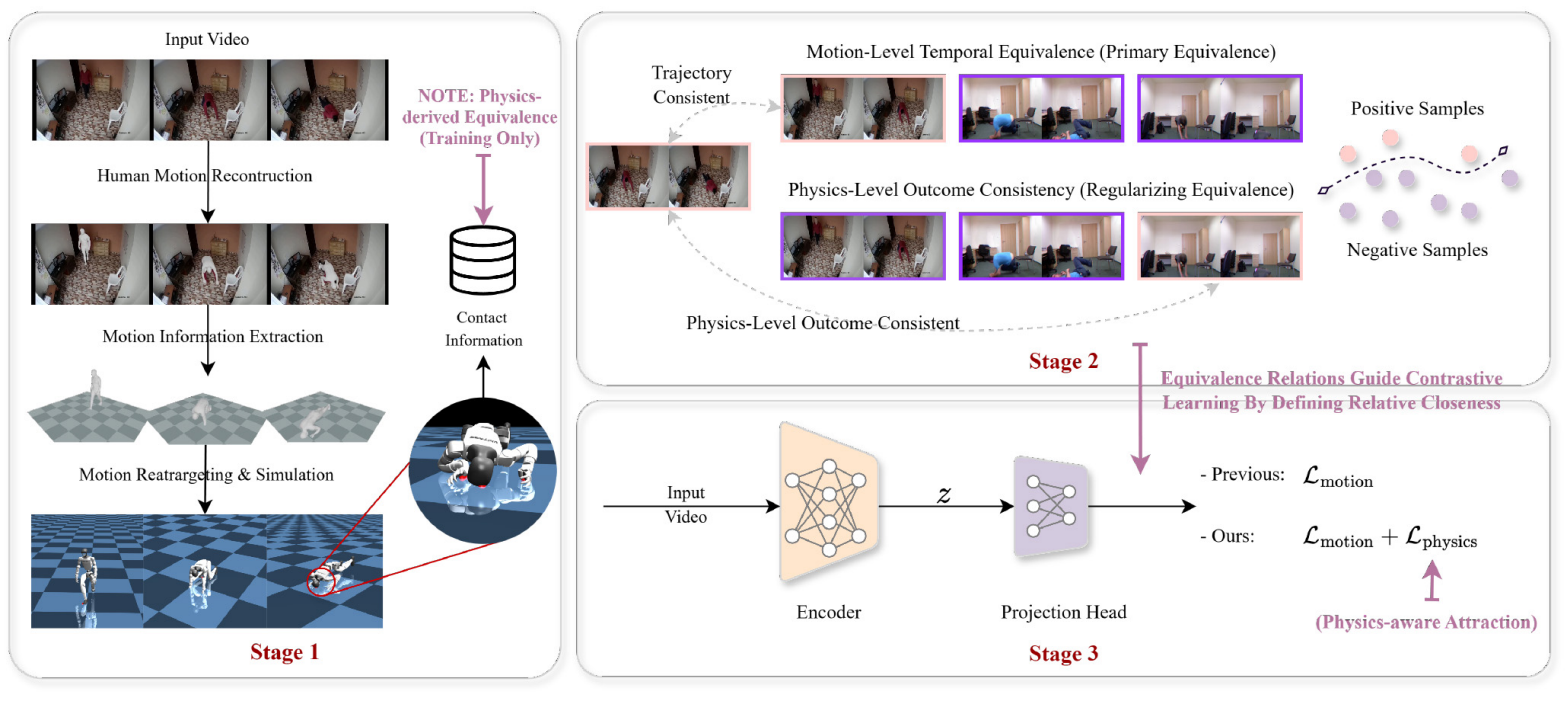
Overview of PHARL. Stage 1 (training only): RGB videos are processed offline to reconstruct motion and run physics simulation, producing window-level contact outcomes (*Supported, Trunk, Head*). Stage 2: PHARL applies two complementary constraints, namely trajectory-consistent temporal positives and physics-level contact structure across trajectories. Physics structure is used for both denominator masking in the trajectory loss and auxiliary same-class attraction among contact windows. Stage 3: An encoder is trained with a composite objective (Equation 3) that improves contact-aware geometry without adding an outcome-prediction head. During inference, PHARL uses only the feed-forward RGB encoder, with no physics simulation.

Accordingly, PHARL can be interpreted as a redesign of contrastive relations, in which physics-aligned attraction enriches the positive structure and denominator masking removes physically inappropriate negatives. The PHARL framework contains three components (Figure 1): (1) a motion representation encoder, instantiated as a physics-free baseline and a physics-regularized variant under the same architecture; (2) a physics module that converts reconstructed motion into window-level contact outcomes and relation metadata; (3) learning objectives that jointly enforce temporal coherence and physics-level outcome consistency. In objective-design terms, temporal consistency primarily constrains local intra-trajectory smoothness, whereas physics alignment primarily constrains cross-trajectory contact-aware structure; together, they reduce motion-outcome aliasing rather than fit an explicit ordinal target.

Both encoder variants share the same backbone, so performance differences arise from supervision design rather than model capacity. The baseline is trained with motion-level consistency only, whereas the PHARL variant adds physics-guided regularization. This paired design isolates the effect of supervision under fixed encoder capacity, allowing differences in geometry and ordinal diagnostics to be attributed to the learning objective rather than model size. Physics information is required only during training; at inference, both models run as standard feed-forward RGB encoders without simulation overhead.

**Boundary clarification**. (1) Input: PHARL takes raw RGB clips as model input. (2) Training-only metadata: 3D human pose/SMPL estimates are extracted offline for simulation and relation construction, and are never fed into the encoder. (3) Supervision type: Physics labels are weak structural signals (for geometric constraints), not pseudo-label targets for an outcome prediction head. (4) Terminology: We distinguish between contacts observed within the current window and contacts observed from a short-horizon continuation initialized at the window boundary. Both are mapped to the same coarse outcome categories *y*^phys^∈{*Supported, Trunk, Head*}. Throughout the manuscript, “outcome” refers to these physics-defined contact categories (not clinical injury severity). The short-horizon continuation is included to capture immediate post-boundary impacts that may not yet appear in the window itself.

### 3.3 Motion-level temporal consistency

We use a trajectory-based contrastive objective to preserve motion coherence. For anchor embedding **z**_*i*_, positives are other views/windows from the same trajectory, while samples from other trajectories serve as contrastive negatives. The motion term is defined in Equation 1.


$$\begin{array}{l} {ℒ}_{\text{motion}}=-\log\frac{\underset{j\in P_{i}^{\text{traj}}}{\sum }\exp\;\mathrm{sim}\left(z_{i},z_{j}\right)/\tau \;}{\underset{k\in A_{i}}{\sum }\exp\;\mathrm{sim}\left(z_{i},z_{k}\right)/\tau \;}, \end{array}$$ (1)

where $z_{i}\in {\mathbb{R}}^{d}$ is the ℓ_2_-normalized embedding of anchor *i*, sim(·, ·) denotes cosine similarity, τ is the motion-branch temperature, $P_{i}^{\text{traj}}$ is the trajectory-positive set for anchor *i*, and $A_{i}$ is the candidate set excluding self-comparisons.

This term stabilizes temporal representation learning, but it is insufficient for contact-state awareness: clips from different trajectories may appear visually similar while leading to different contact consequences. PHARL addresses this limitation by injecting physics-derived structure into training. In essence, the motion objective provides a temporal prior, and the physics objective provides structural correction. PHARL therefore refines motion representation rather than replacing it with outcome prediction.

### 3.4 Physics-level outcome consistency

#### 3.4.1 Outcome structure as weak supervision

This module injects physics-consistent structure into the embedding space without introducing an outcome classifier. Each window receives a coarse physics label *y*^phys^∈{*Supported, Trunk, Head*}, which is used only to define geometric constraints in contrastive learning as defined in Equation 2. PHARL uses this supervision in two complementary ways: (1) binary contact grouping (*Head/Trunk* vs. *Supported*) for denominator masking, and (2) exact class matching (*Trunk-to-Trunk, Head-to-Head*) for auxiliary attraction. The first reduces harmful repulsion among contact windows; the second strengthens cross-trajectory alignment for contact-consistent events. Together, they increase outcome-aware organization under weak supervision while preserving feed-forward RGB inference.

#### 3.4.2 Outcome extraction and denoising

Physics-derived descriptors are used as weak structural supervision rather than prediction targets. The purpose of denoising is to resolve the *visual-semantic gap*—a fundamental source of noise where temporally mismatched labels contaminate the representation. For instance, in traditional trajectory-level labeling, every window of a fall video inherits the same label, forcing the encoder to associate pre-impact standing poses with severe outcomes (e.g., “Head Impact”). PHARL's denoising process produces labels that are temporally aligned with each window and robust to transient simulation noise.

For each video window $W=\left[t_{0},t_{1}\right)$, label construction follows three steps. First, temporal alignment retains only contact descriptors whose interval [*t*_*s*_, *t*_*e*_) overlaps W, i.e., (*t*_*s*_<*t*_1_)∧(*t*_*e*_>*t*_0_). This step correctly re-assigns pre-impact frames to the *Supported* class, ensuring that severe labels are reserved for actual impact kinematics. Second, boundary completion incorporates short-horizon continuation evidence from the window endpoint, so that impacts that occur immediately after *t*_1_ can still inform the current state. This step addresses boundary truncation in fixed-length windows, where pre-impact frames and impact onset may be split across adjacent windows. Third, reliability filtering suppresses weak impulses and aggregates the remaining evidence by taking the category-wise maximum impulse across available sources. These operations define a deterministic, reproducible mapping from raw contact descriptors to window-level supervision.

Let the filtered impulse set for W be $S_{W}$. The coarse physics label is assigned by hierarchical dominance: This priority rule is physics-motivated: head contact is treated as the most critical outcome state, followed by trunk contact, while all remaining cases are mapped to *Supported*.


$$\begin{array}{l} y^{\text{phys }}(W)=\{\begin{array}{ll} Head\text{, } & \text{ if head-contact impulse is present }\\ \; & \text{ in }S_{W},\\ Trunk\text{, } & \text{ if torso/hip-contact impulse is present }\\ \; & \text{ in }S_{W},\\ Supported\text{, } & \text{ otherwise (including arm/hand/leg/ }\\ \; & \text{ foot contacts). } \end{array} \end{array}$$ (2)

If no valid overlapped descriptor is available (or the window is non-fall), the label defaults to *Supported*.

In this context, denoising provides stable weak supervision for physics regularization without introducing an explicit outcome-prediction head. It ensures the representation geometry is shaped by temporally localized physical evidence rather than coarse video-level proxies.

#### 3.4.3 Representation learning objectives

The physics-regularized encoder combines trajectory consistency with outcome-aware regularization. The loss function is defined in Equation 3.


$$\begin{array}{l} ℒ={ℒ}_{\text{motion}}+\lambda_{\text{phys}}{ℒ}_{\text{physics}}+\lambda_{\text{var}}{ℒ}_{\text{var}}. \end{array}$$ (3)

Here, ${ℒ}_{\text{motion}}$ is the trajectory-consistency term, ${ℒ}_{\text{physics}}$ is the physics-alignment term, and ${ℒ}_{\text{var}}$ is the variance regularizer that prevents representation collapse. λ_phys_ and λ_var_ are balancing coefficients.

For denominator masking, we define a binary contact indicator $C_{i}=1\left[y_{i}^{\text{phys}}\in \left\{\text{Head, Trunk}\right\}\right]$ and the marked set is shown in Equation 4.


$$\begin{array}{l} {ℳ}_{i}=\left\{k\in A_{i}|C_{i}=1,C_{k}=1,\text{traj}\left(k\right)\neq \text{traj}\left(i\right)\right\}, \end{array}$$ (4)

where *C*_*i*_ is the binary contact indicator for anchor *i* and ${ℳ}_{i}$ is the masked candidate set that removes cross-trajectory contact windows from the denominator. If *C*_*i*_ = 0 (*Supported* anchor), then ${ℳ}_{i}=\emptyset$. The masked trajectory loss is defined in Equation 5.


$$\begin{array}{l} {ℒ}_{\text{motion}}=-\log\frac{\underset{j\in P_{i}^{\text{traj}}}{\sum }\exp\left(\mathrm{sim}\left(z_{i},z_{j}\right)/\tau \right)}{\underset{k\in A_{i}\backslash {ℳ}_{i}}{\sum }\exp\left(\mathrm{sim}\left(z_{i},z_{k}\right)/\tau \right)}, \end{array}$$ (5)

where denominator candidates are restricted to $A_{i}\backslash {ℳ}_{i}$, i.e., all available comparisons after masking. Without this masking step, contact windows from different trajectories can be treated as hard negatives, which introduces false repulsion and weakens contact-consistent geometry.

For auxiliary attraction, contact anchors use exact class matching across trajectories, as shown in Equation 6.


$$\begin{array}{l} P_{i}^{phys}=\left\{j|y_{j}^{\text{phys}}=y_{i}^{\text{phys}},y_{i}^{\text{phys}}\in \left\{\text{Head, Trunk}\right\},\text{traj}\left(j\right)\neq \text{traj}\left(i\right)\right\}. \end{array}$$ (6)

Here, $P_{i}^{phys}$ denotes same-class (*Head, Trunk*) cross-trajectory positives for anchor *i*, and *Q*_*i*_ = {*k*∣traj(*k*)≠traj(*i*)} denotes all cross-trajectory candidates for anchor *i*. The auxiliary term is defined in Equation 7.


$$\begin{array}{l} {ℒ}_{\text{physics}}=-\log\frac{\underset{j\in P_{i}^{phys}}{\sum }\exp\left(\mathrm{sim}\left(z_{i},z_{j}\right)/\tau_{p}\right)}{\underset{k\in Q_{i}}{\sum }\exp\left(\mathrm{sim}\left(z_{i},z_{k}\right)/\tau_{p}\right)}, \end{array}$$ (7)

where τ_*p*_ is the physics-branch temperature. Without this exact-class attraction term, the embedding tends to preserve coarse contact/non-contact separation but under-represents fine-grained ordinal structure between *Trunk* and *Head*.

While the scarcity of severe events (*Head*) often leads to their merging with *Trunk* into a single binary category, we explicitly enforce exact multi-class attraction. This design choice is important for revealing the emergent ordinal organization of the manifold; by pulling only exact matches together, the model is encouraged to learn distinct impact-relevant kinematic signatures associated with different contact outcomes. In practice, anchors with empty positive sets (e.g., no available cross-trajectory same-class partner) are skipped for the corresponding term, following standard contrastive training conventions.

This formulation encourages contact-consistent geometry without introducing an outcome prediction head or ordinal supervision. Each term serves a specific role: ${ℒ}_{\text{motion}}$ (with masking) preserves trajectory-level temporal consistency while reducing unnecessary repulsion among contact windows; ${ℒ}_{\text{physics}}$ strengthens cross-trajectory alignment for matched outcome classes (*Head, Trunk*); and ${ℒ}_{\text{var}}$ maintains embedding spread to prevent collapse.

### 3.5 Training and inference workflow

For clarity, we summarize PHARL training as a four-step pipeline (input/output view). (1) Window construction: each trajectory is segmented into overlapping windows (input: RGB trajectory; output: window set). (2) Physics signal extraction (training only): each window is associated with simulation-derived contact outcomes and contact statistics, with short-horizon continuation used to recover immediate post-boundary contacts (output: window-level physics metadata). (3) Relation building: trajectory-positive sets, denominator masks, and cross-trajectory same-class sets are built from window metadata (output: contrastive relation graph). (4) Joint optimization: the encoder is updated with Equation 3, combining temporal consistency and physics-alignment regularization (output: trained RGB encoder).

Algorithmically, the key distinction from standard temporal contrastive learning is that negative/positive relations are no longer determined only by trajectory identity or augmentation pairing. PHARL uses physics equivalence to suppress harmful negatives (contact-contact false repulsion) and to add structured positives (same-class cross-trajectory attraction), thereby improving outcome-aware geometry under weak supervision.

At inference, the pipeline is simplified to a single feed-forward RGB encoder; simulation, contact extraction, and relation construction are all removed. Therefore, PHARL preserves deployment efficiency while retaining the physically informed structure learned during training.

## 4 Experiments

### 4.1 Datasets

We evaluate PHARL on four public fall datasets and aggregate them into a unified benchmark spanning multiple scenes, viewpoints, and subjects. Table 1 summarizes the resulting dataset profile, including trajectory counts and available subject/scene/camera metadata relevant to bias analysis.

**Table 1:** Dataset profile.

Dataset	Videos	Fall	ADL	Subjects	Scenes	Cameras
CAUCAFall (20)	100	50	50	10	–	–
GMDCSA-24 (39)	160	79	81	4	–	–
Le2i (17)	190	130	60	–	6	–
URFD (18)	100	60	40	–	–	2

This table summarizes the four public benchmarks used in this work. Subject, scene, and camera counts are reported only when these fields are explicit in the dataset metadata.

Le2i (17) contains 190 indoor sequences. URFD (18) provides 100 sequences in our statistics: 40 fall videos and 60 ADL videos, where the ADL subset counts both camera views (cam0 and cam1; 30 per view). CAUCAFall (20) includes 100 home-environment sequences captured from multiple camera angles. GMDCSA-24 (39) contributes 160 trajectories with diverse fall directions and protective responses. This profile makes several bias sources explicit. Subject coverage is limited in CAUCAFall (10 subjects) and especially GMDCSA-24 (4 subjects), Le2i concentrates scene diversity into six indoor environments without explicit subject metadata, and URFD contains only two camera views. A lightweight frame-level summary further indicates substantial visual heterogeneity across datasets: CAUCAFall is darker on average (mean luminance 88.7), GMDCSA-24 has the highest nominal resolution (1280 × 720), Le2i has the lowest nominal resolution (320 × 240), and URFD shows the largest luminance variability (standard deviation 37.3). These differences motivate both the mixed-dataset evaluation and the Leave-One-Dataset-Out transfer protocol in Section 4.8.

None of the datasets provides clinical injury annotations. Binary fall labels are used only for aggregation and baseline diagnostics, not as supervision for physics-regularized representation learning. We perform trajectory-level train/val/test splitting to avoid window leakage, with stratified sampling to preserve physics-outcome diversity across splits. The final split is 438/56/56 trajectories (train/val/test). Physics contact descriptors are precomputed and cached per video, and outcome tags from these descriptors are used only for split stratification. This protocol enforces strict trajectory-level separation throughout the pipeline.

Each video is converted into overlapping windows using a sliding-window strategy. For each window, we derive a coarse physics outcome label *y*^phys^ ∈ {*Supported, Trunk, Head*} from contact statistics. These labels are used only for training-time regularization and *post-hoc* evaluation, never as encoder input or prediction targets. Window labels are computed with a minimum impulse threshold of 0.0 N·s; video and physics windows are aligned by temporal overlap. Depending on the ablation setting, labels are derived from in-window contacts, short-horizon continuation contacts, or their union; when both are used, sources are merged by per-category maximum impulse. The training split is imbalanced (*Supported* ≈56%, *Trunk* ≈34%, *Head* ≈10%), with similar class trends on validation and test sets.

### 4.2 Evaluation metrics

We design an evaluation suite to test whether the learned embedding geometry is consistent with physics-derived outcomes , without using clinical injury labels. Accordingly, all probe-based metrics are treated as diagnostics of representation quality rather than as stand-alone clinical predictors. All reported metrics are *post-hoc* diagnostics. Model checkpoints are selected solely based on the minimum validation loss under the training objective. Throughout this section, “outcome” refers to physics-derived contact categories (*Supported, Trunk, Head*). These categories are analysis labels, not optimization targets.

We define a *post-hoc* severity axis after training using train-split centroids as shown in Equation 8.


$$\begin{array}{l} \begin{array}{cc} \hat{v} & =\frac{\mu_{\text{Head}}-\mu_{\text{Supported}}}{\mu_{\text{Head}}-\mu_{\text{Supported}}}\;\text{(severity axis)},\\ s_{i} & =z_{i}^{⊤}\hat{v}\;\text{(projection score)}. \end{array} \end{array}$$ (8)

Here, ***μ**_Head_* and ***μ**_Supported_* denote class centroids (computed on the training set), and *s*_*i*_ denotes the scalar projection of embedding **z**_*i*_ onto the *post-hoc* severity axis. This projection score is used only for *post-hoc* analysis and is not a prediction output. Using ordinal labels {0, 1, 2} for {*Supported, Trunk, Head*}, we report metrics in the following priority order:

1. Spearman's ρ: rank correlation between ordinal labels and projection scores *s*_*i*_, computed on their respective ranks, reflecting global monotonic alignment along the *post-hoc* severity axis.
2. POA (Macro): macro-averaged pairwise ordering accuracy over sampled instance pairs, measuring whether higher-severity outcomes are consistently ranked above lower-severity ones.
3. Binary Contact AP: average precision of a linear probe distinguishing contact (*Head, Trunk*) from *Supported* cases, emphasized under class imbalance.
4. Binary Contact AUC: ROC-AUC of the same linear probe, capturing threshold-free separability between contact and *Supported* instances.
5. Fall Detection AUC: ROC-AUC of a linear probe for fall vs. no-fall, reported solely as a deployment-oriented sanity check.
6. Physics Consistency Ratio (PCR): ratio of inter-class to intra-class embedding distances defined by *y*^phys^, indicating geometry-level separation consistent with physics-derived categories.
7. Kendall's τ: concordance-based rank correlation between ordinal labels and projection scores, reported as a robustness check for ordinal trends.

Accordingly, Spearman's ρ and POA serve as the primary evidence of ordinal consistency, Binary Contact AP/AUC quantify contact-localization utility, and Fall Detection AUC, PCR, and Kendall's τ provide secondary diagnostics of deployment sanity and geometry quality.

### 4.3 Baselines

We compare PHARL against six baselines using identical data splits. All methods rely on raw RGB clips with trajectory-consistent temporal positives for stable optimization. Only PHARL incorporates physics-derived structural supervision. This deliberate information asymmetry isolates the effect of physics regularization, rather than asserting superiority under equivalent supervision.

- Vanilla InfoNCE (25): A standard contrastive learning baseline optimized with the InfoNCE objective, without auxiliary losses or structural constraints.
- Hard Negative Mining (HNM) (26): A contrastive variant that re-weights or emphasizes hard negative samples (i.e., negatives most similar to the anchor) to encourage finer-grained separation.
- Barlow Twins (BT) (40): A redundancy-reduction method that enforces decorrelation between embedding dimensions via a cross-correlation objective.
- SimSiam (41): A negative-free baseline based on asymmetric prediction and stop-gradient. In our implementation, each training item uses two augmented views of the anchor window and one additional motion-equivalent view sampled from a coarse temporal-phase bucket (early/mid/late).
- Contrastive Disentangling (CD) (27): A multi-head contrastive framework designed to disentangle latent factors, with auxiliary constraints promoting feature diversity.
- Multi-Grained Contrast (MGC) (37): A temporal contrastive approach that aligns local window embeddings with a global trajectory representation to capture multi-scale temporal structure.

### 4.4 Implementation details

We use a ResNet-18 encoder with temporal average pooling and a projection head to produce ℓ_2_-normalized embeddings. Physics simulation is run offline only to derive contact descriptors; physics labels are never provided as encoder inputs.

All models are trained for 100 epochs. We select the primary checkpoint by minimum validation loss and additionally report final-epoch checkpoints. Baseline and PHARL runs share learning rate 1 × 10^−4^ and trajectory-contrast temperature τ = 0.2. PHARL further uses an auxiliary physics temperature of 0.2 with warmup-based physics-weight scheduling. A shared memory bank is used to stabilize contrastive optimization, and physics-stratified sampling is enabled only for PHARL variants that use physics labels. In summary, architecture, data splits, learning rate, and base temperature are shared across methods, while physics-specific settings are applied only to PHARL.

### 4.5 Performance comparison

Table 2 reports a quantitative comparison between PHARL and representative self-supervised baselines. To facilitate comparison with motion-only alternatives, we use the strongest such baseline (MGC) as the reference point and report PHARL's additional gains as evidence that physics-guided relation design contributes beyond motion-context enrichment alone. All comparisons are interpreted within a common probing protocol as diagnostics of representation quality, without altering the non-predictive training formulation. The key finding is the emergence of a clear ordinal structure in the learned representation space. PHARL achieves a Spearman ρ of 0.4800 and a POA of 0.7983, substantially outperforming all motion-only alternatives and indicating strong alignment between latent geometry and the physics-derived risk ordering.

**Table 2:** Performance comparison.

Method	Spearman ρ	POA (Macro)	Binary contact		Fall detection AUC	PCR	Kendall τ
			AP	AUC			
Vanilla	0.2232	0.6221	0.4992	0.7056	0.7736	1.0082	0.1768
HNM	0.2454	0.6234	0.5979	0.7762	0.8081	1.0032	0.1951
BT	0.2405	0.6190	0.5979	0.7688	0.8289	1.0055	0.1911
SimSiam	0.0396	0.5199	0.3792	0.5819	0.5667	**1.0390**	0.0315
CD	0.2189	0.6255	0.5429	0.7476	0.8302	1.0076	0.1736
MGC	0.2754	0.6382	0.6452	0.7987	0.8405	1.0046	0.2189
PHARL	**0.4800**	**0.7983**	**0.6484**	**0.8223**	**0.8996**	1.0126	**0.3850**

This table reports the quantitative comparison across methods. Bold indicates the best result and underlined values indicate the second-best result.

Standard contrastive objectives, including Vanilla InfoNCE, HNM, and BT, show limited ability to capture such ordinal relationships. Although these methods improve local feature discrimination and moderately increase Binary Contact AUC (e.g., 0.7762 for HNM and 0.7688 for BT), their objectives remain primarily instance-driven and provide no structural mechanism for organizing motion patterns by physical outcomes. Consequently, the learned embeddings exhibit weak ordinal organization, with Spearman ρ values below 0.25.

Among the motion-driven baselines, MGC is the strongest. MGC improves ordinal correlation to ρ = 0.2754 and achieves a Fall Detection AUC of 0.8405. PHARL further increases ordinal correlation to 0.4800, representing a substantial gain in ordinal alignment. This comparison indicates that richer motion context alone is insufficient to resolve the ambiguity of fall dynamics, whereas physics-outcome structure provides stronger guidance for representation organization.

CD adopts a multi-head representation-learning strategy but shows limited benefits in this highly imbalanced fall scenario. Its ordinal metrics remain comparable to those of Vanilla InfoNCE, suggesting that disentangling visual factors without physical grounding does not naturally isolate features associated with impact severity.

Finally, SimSiam exhibits clear limitations in this safety-critical setting, yielding a near-zero Spearman ρ of 0.0396. Although it attains a relatively high PCR, the absence of negative pairs weakens the discriminative pressure needed to resolve fine-grained severity differences, resulting in representations that lack meaningful ordinal structure.

Overall, these results demonstrate that PHARL effectively mitigates the kinematic ambiguity inherent in fall motions. By introducing physics-outcome consistency as a structural regularizer during representation learning, PHARL improves both ordinal risk alignment (Spearman, Kendall, and POA) and safety-localization performance over purely motion-driven self-supervised objectives.

### 4.6 Ablation studies

We conduct ablation studies to evaluate the contribution of three key components of PHARL: the denoising mechanism for window-level alignment, the multi-class attraction module for ordinally structured contrastive learning, and the two sources of physics-derived supervision (window-level contact and outcome-based continuation). Results are summarized in Table 3. These ablations isolate three functional roles: denoising improves temporal label fidelity, multi-class attraction refines class-specific structure, and the two supervision sources balance local impact cues with longer-horizon outcome organization. The first row reports a temporal-only control in which all physics-specific components are removed, while the encoder, optimization schedule, and trajectory-consistency objective are kept unchanged.

**Table 3:** Ablation study of PHARL components.

Denoising	Multi-class	Physics information		Spearman ρ	POA (Macro)	Binary contact		Fall detection AUC	PCR	Kendall τ
		Window	Continuation			AP	AUC			
×	×	×	×	0.2224	0.6589	0.6002	0.7606	0.8343	1.0065	0.1767
×	✓	✓	✓	0.3716	0.7199	0.5516	0.7747	0.8701	0.9873	0.2974
✓	×	✓	✓	0.4282	0.7685	0.6523	0.8249	0.8745	1.0070	0.3413
✓	✓	×	✓	0.4708	0.7850	0.6085	0.8146	**0.9088**	**1.0180**	0.3774
✓	✓	✓	×	0.3756	0.7272	**0.7051**	**0.8339**	0.8960	1.0179	0.2990
✓	✓	✓	✓	**0.4800**	**0.7983**	0.6484	0.8223	0.8996	1.0126	**0.3850**

This table reports the effect of each component. Bold indicates the best result and underlined values indicate the second-best result.

This control shows that temporal consistency alone does not recover the same ordered geometry. Relative to full PHARL, Spearman ρ decreases from 0.4800 to 0.2224, POA from 0.7983 to 0.6589, and Binary Contact AP from 0.6484 to 0.6002. These results indicate that the observed ordinal structure arises from physics-aware relation design rather than from the backbone or motion objective alone.

Removing the denoising mechanism leads to a substantial degradation in ordinal consistency. When trajectory-level supervision is used directly while the remaining physics components are retained (second row), Spearman ρ decreases from 0.4800 to 0.3716 and Binary Contact AP drops by nearly 10% (0.6484–0.5516). This degradation indicates that coarse trajectory labels introduce significant label noise, where pre-impact upright frames become associated with high-severity outcomes. The proposed denoising step mitigates this mismatch by aligning supervision with temporally localized motion segments. Denoising removes temporally inconsistent contact assignments and preserves supervision aligned with near-impact windows, thereby reducing leakage from pre-impact frames. It therefore functions as a temporal alignment step that reconciles trajectory-level tags with window-level motion evidence.

The multi-class attraction mechanism also plays an important role in shaping the severity ordering of the learned representations. Replacing the exact class-based attraction with binary grouping (*Head* and *Trunk* treated as the same class) reduces Spearman ρ from 0.4800 to 0.4282 and Kendall's τ from 0.3850 to 0.3413. While binary supervision remains sufficient to distinguish impact events from benign activities, it fails to preserve the relative ordering among different impact severities, highlighting the importance of class-specific contrastive attraction.

The subset ablations further reveal a trade-off between instantaneous impact detection and global outcome alignment. Training with window-level supervision alone yields the strongest Binary Contact performance, achieving the highest AUC (0.8339) and AP (0.7051), as the supervision signal directly corresponds to observable impact frames. However, this localized training signal results in weaker ordinal structure, with Spearman ρ dropping to 0.3756. In contrast, continuation-only supervision improves the global ordering of trajectories (Spearman ρ of 0.4708) but slightly reduces contact localization performance.

Combining denoising, multi-class attraction, and both sources of supervision yields the most balanced overall results. Overall, the ablations indicate that temporal denoising primarily improves supervision fidelity, class-specific attraction sharpens ordinal separation, and the combination with dual-source supervision provides the best balance between contact localization and ordinal consistency. The full PHARL configuration achieves the highest ordinal consistency (Spearman ρ of 0.4800, POA of 0.7983, and Kendall's τ of 0.3850) while maintaining competitive fall detection performance (AUC of 0.8996). These results suggest that motion-level temporal alignment and physics-outcome consistency provide complementary supervision signals for learning risk-aware representations.

### 4.7 Representation analysis

**Figure 2:**
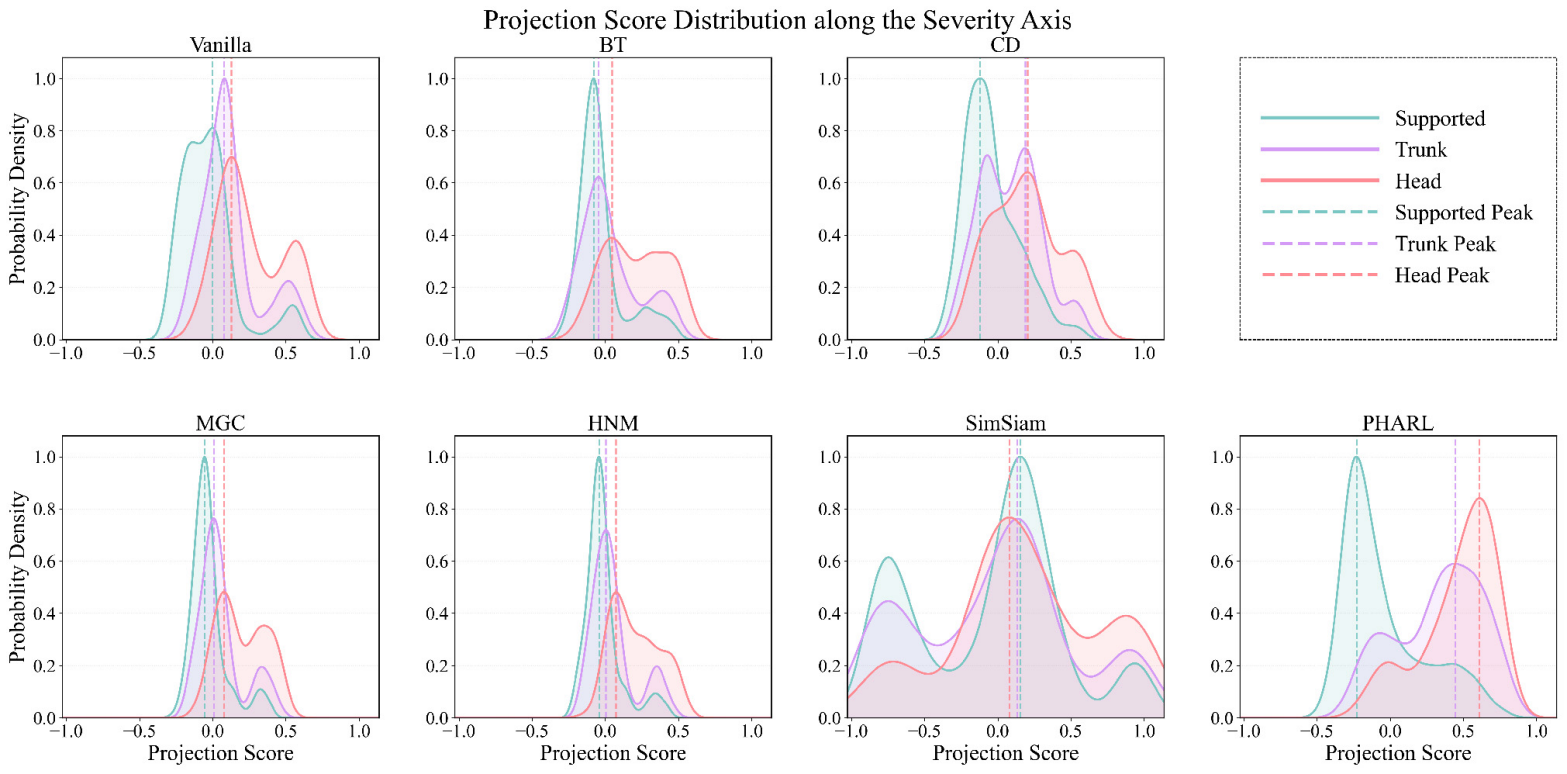
Kernel density estimates of embedding distributions along the *post-hoc* severity axis (defined by the *Supported* and *Head* class centroids). Baseline methods show substantial class overlap, whereas PHARL exhibits a clearer staircase-like separation. This pattern indicates improved alignment between latent geometry and physics-consistent impact outcomes. The projection is used for analysis only and is not a prediction output.

**Figure 4:**
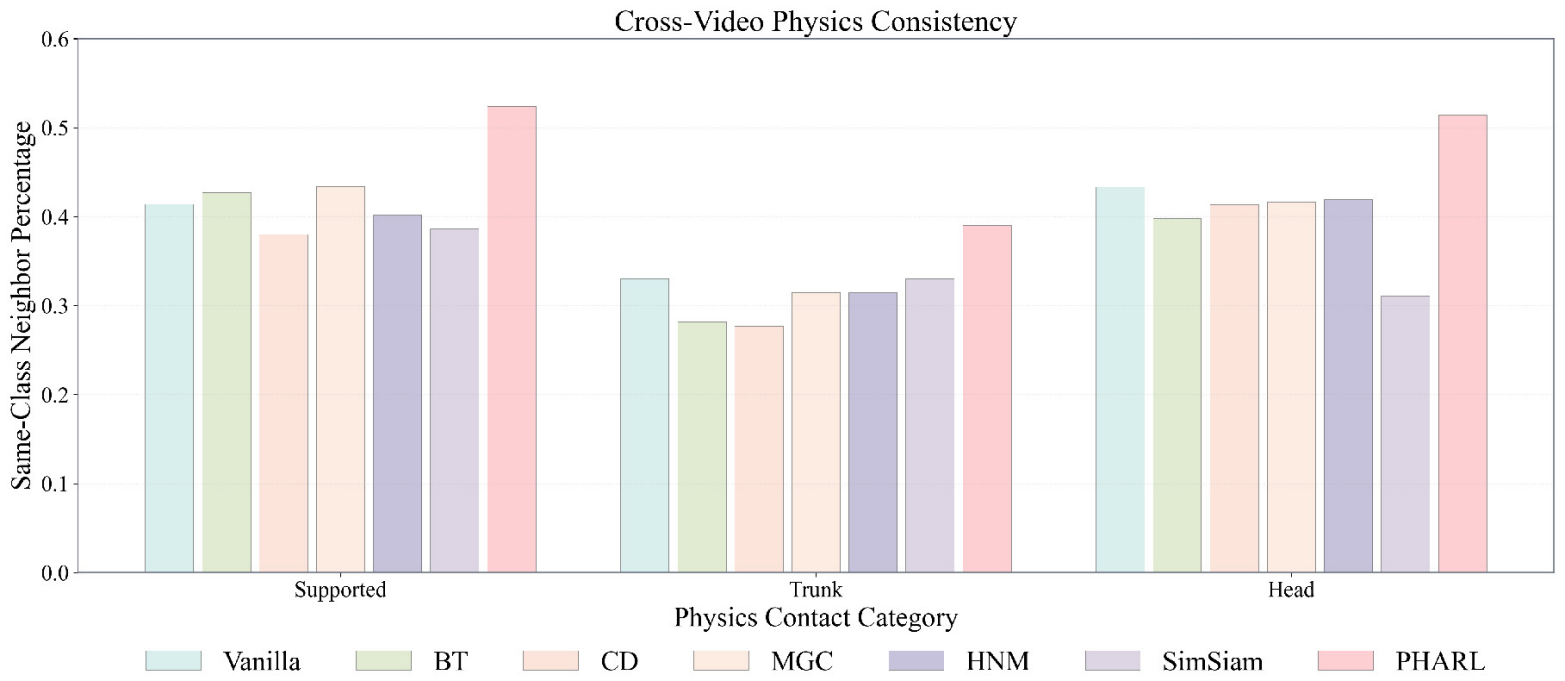
Cross-video neighborhood consistency across physics contact categories. For each split, a class-balanced retrieval database is built, queries include all windows per class, and cosine nearest neighbors are retrieved after excluding same-video samples. Bars report row-normalized diagonal consistency (*Supported@Supported, Trunk@Trunk, Head@Head*) with *k* = 10. PHARL consistently achieves higher same-class consistency, indicating stronger cross-video physical structure in the learned representation.

**Figure 3:**
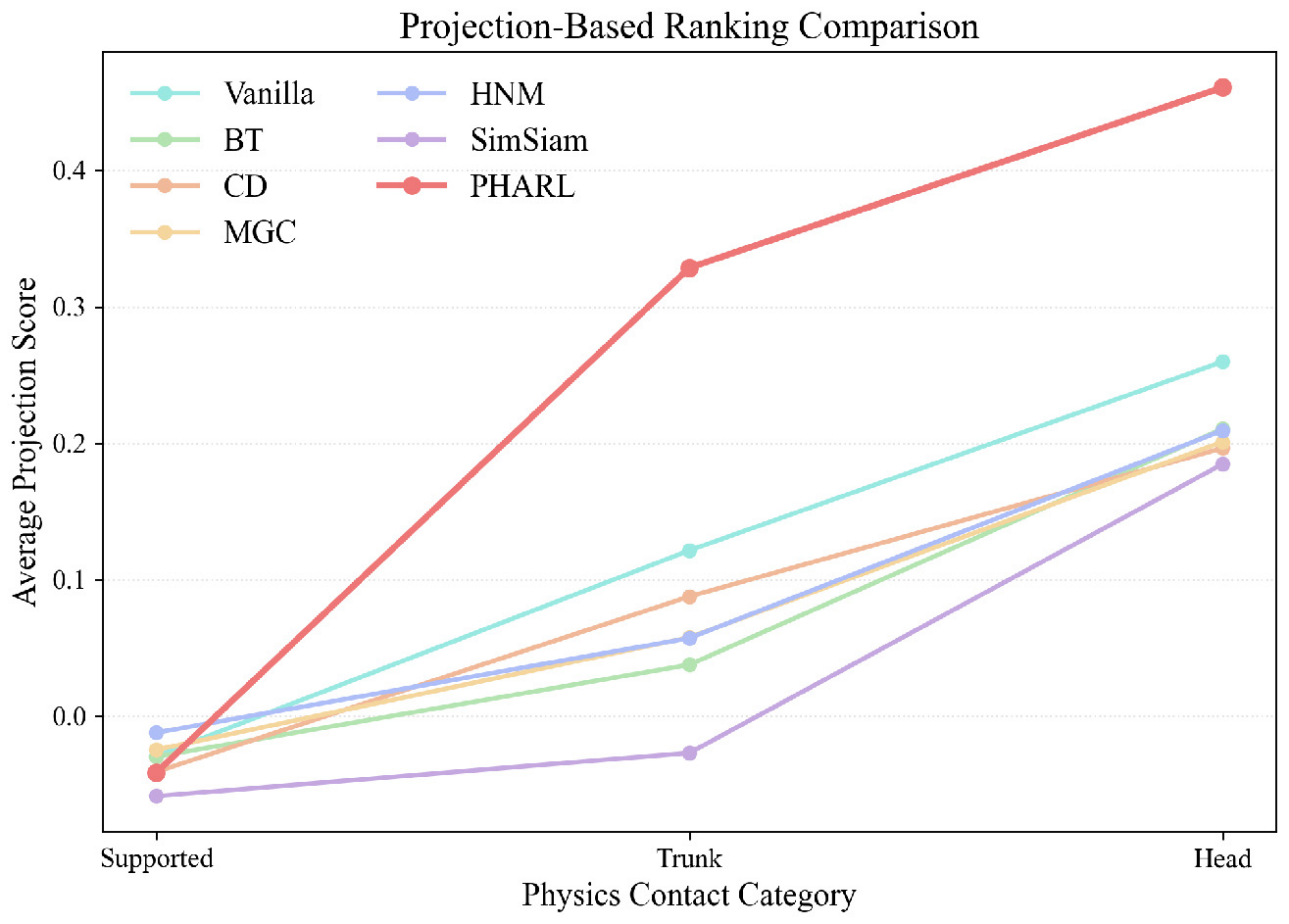
Category-wise mean projection scores along the *post-hoc* severity axis across contact outcomes. PHARL preserves the expected physical ordering (*Head* ≻ *Trunk* ≻ *Supported*) with a larger margin than motion-only baselines, indicating stronger ordinal organization in the learned representation. These projection scores are descriptive diagnostics and should not be interpreted as severity predictions.

To further examine the structural properties of the learned representation, we conduct three complementary manifold analyses focused on distribution separation, ordinal consistency, and cross-video neighborhood structure. These analyses provide qualitative evidence of how different methods organize impact-related motions in latent space. In particular, they test whether physics-regularized training induces a geometry that is consistent with the underlying ordering of physical impact outcomes. Mechanistically, temporal consistency preserves short-horizon semantic continuity within trajectories, whereas physics alignment imposes cross-trajectory structure among windows with similar contact outcomes. Their interaction reduces kinematic-outcome ambiguity and supports an emergent ordinal organization in latent space. Figures 2–4 therefore provide complementary visualization evidence for distribution overlap, category-wise ordering, and cross-video physical neighborhood structure. Within this geometry, the most informative cues are expected to be impact-localized motion patterns associated with contact configuration, including protective upper-limb bracing, torso-first support, head-ground proximity at impact, and temporally concentrated deceleration near contact. Because PHARL is optimized to shape representation geometry rather than to produce attribution maps, these cues are interpreted through manifold structure and cross-video neighborhood relations rather than through pixel-level saliency.

Figure 2 visualizes the density of embeddings projected onto a *post-hoc* severity axis. This axis is defined as the unit vector from the *Supported* centroid to the *Head* centroid in latent space, yielding a physically interpretable direction that roughly corresponds to increasing impact severity. Importantly, this axis is used only for *post-hoc* visualization and does not affect training or quantitative metrics. Whereas baseline methods show substantial overlap among category distributions, PHARL produces a more structured manifold with staircase-like ordinal separation along this direction. Specifically, the *Head* and *Trunk* distributions shift rightward relative to *Supported*, suggesting that the learned representation resolves kinematic ambiguity and organizes motions according to physics-consistent impact outcomes.

The emergence of ordinal structure is further examined in Figure 3, which reports the mean projection score of each category along the same severity axis. PHARL consistently preserves the expected physical hierarchy (*Head* ≻ *Trunk* ≻ *Supported*) with a larger margin than motion-only baselines. This monotonic pattern indicates that physics-regularized alignment helps the encoder recover the ordinal structure of fall severity as an emergent property, despite the absence of explicit ranking supervision during training. Because these statistics are computed at the category level, the observed ordering is relatively insensitive to sample-count differences across categories.

Finally, we assess whether embeddings capture physical identity beyond individual video instances via cross-video neighborhood analysis (Figure 4). For each split, we build a class-balanced retrieval database by subsampling *Supported, Trunk*, and *Head* windows to a common size. Queries are drawn from all windows in each class, and nearest neighbors are retrieved using cosine similarity in embedding space. To reduce video-specific shortcuts, same-video neighbors are excluded before selecting up to top-*k* valid neighbors (*k* = 10). PHARL yields higher diagonal consistency across impact categories than baseline methods. This suggests that the representation captures generalized physical characteristics associated with impact outcomes, rather than relying mainly on appearance cues or video-level context. Taken together, these analyses indicate that PHARL learns a latent geometry aligned with both ordinal structure and physical identity of impact outcomes.

### 4.8 Cross-dataset generalization under a Leave-One-Dataset-Out protocol

To assess cross-dataset transfer more directly, we evaluate PHARL under a Leave-One-Dataset-Out (LODO) protocol. For each target dataset, all trajectories from that dataset are held out for testing, and the remaining datasets are used to construct source-domain training/validation splits. This setting introduces a full domain shift in viewpoint, scene context, and capture protocol, and thus provides a stricter transfer test than the mixed-dataset split in Tables 2, 3.

As summarized in Table 4, PHARL improves ordinal metrics on all four held-out datasets, but the effect size is domain-dependent. CAUCAFall shows only modest changes, including nearly unchanged Contact AP/AUC and no gain in Fall Detection AUC, whereas clearer improvements are observed on GMDCSA-24, Le2i, and URFD.

**Table 4:** LODO cross-dataset evaluation.

Held-out dataset	Method	Spearman ρ	POA	Contact AP	Contact AUC	Fall Detection AUC
CAUCAFall	Temporal-only	0.0758	0.5338	0.3451	0.5645	0.6045
	PHARL	0.1079	0.5618	0.3542	0.5666	0.5521
GMDCSA-24	Temporal-only	0.1607	0.6039	0.3537	0.6444	0.5498
	PHARL	0.2689	0.6805	0.4321	0.7102	0.6243
Le2i	Temporal-only	0.0832	0.5464	0.2921	0.5316	0.4813
	PHARL	0.2357	0.6625	0.4060	0.6835	0.7525
URFD	Temporal-only	–0.0779	0.4555	0.1966	0.3448	0.2629
	PHARL	0.2410	0.6568	0.3706	0.6436	0.7042

For each held-out dataset, the model is trained on the remaining datasets and evaluated on the unseen target dataset. This table reports target-domain test performance under held-out-dataset transfer.

Taken together, these results provide cautious evidence that physics-aware relation design can improve transferability of risk-aligned embedding geometry, while also confirming that residual dataset bias remains a limiting factor under domain shift.

## 5 Discussion

### 5.1 Physics-regularized representations and interpretation

PHARL learns a representation geometry that is organized by physics-consistent outcomes (e.g., contact patterns and motion-level equivalences), rather than by visual or kinematic similarity alone. This behavior indicates that physics consistency guides the encoder toward latent factors that are relevant to contact dynamics, without introducing an explicit outcome-prediction objective. As a result, the learned embedding is useful for downstream analysis tasks such as motion retrieval, low-shot transfer, and expert-assisted review, while intentionally avoiding deployable severity scores or decision outputs. In downstream fall-risk workflows, this embedding can support clinician-in-the-loop retrieval, low-shot screening of high-risk motion patterns, and category-level trend analysis over time. It remains a decision-support representation rather than an autonomous diagnostic output. In embodied elderly-care systems, it may also serve as a perception prior that improves robustness, interpretability, and label efficiency for higher-level monitoring or planning modules.

**Weak Supervision vs. Self-Supervision**. Our formulation is best interpreted as physics-guided weak supervision: simulation provides objective, scalable structural signals that shape embedding geometry without requiring manual injury labels. Throughout this manuscript, the term “outcome” denotes physics-derived contact outcomes (*Supported, Trunk, Head*) rather than clinical severity labels. This setting is particularly attractive in safety-critical domains, where dense labels are expensive, inconsistent, or intrinsically difficult to define.

**Head-Contact Rarity and Its Implications**. Head-contact windows remain a minority class (roughly 10%, depending on split), which increases uncertainty in head-related comparisons and can visually compress class separation in 2D projections because of undersampling. Accordingly, we prioritize ordinality metrics (Spearman/POA), report contact AP/AUC as safety-localization utility, treat fall AUC as a deployment sanity check, and interpret PCR/Kendall together with qualitative plots as diagnostic evidence rather than deployable prediction evidence.

### 5.2 Limitations and future work

**Objective-level ordinal structure**. PHARL does not explicitly optimize an ordinal objective. The *Supported–Trunk–Head* ordering therefore emerges implicitly from contact-aware relation design and is evaluated only *post hoc*. Future work can test whether explicit ranking constraints further strengthen monotonic structure without reframing the method as a direct risk predictor.

**Simulation fidelity and environment factors**. The simulator necessarily abstracts fall dynamics and environment interactions, including compliant surfaces, furniture contact, and protective equipment. Accordingly, the physics signal should be interpreted as relative structural guidance for representation learning rather than as absolute biomechanical ground truth. Future work should examine sensitivity to simulation parameters and extend the framework to richer environment-aware and protective-behavior settings.

**Dataset bias and generalization**. The current framework does not explicitly model viewpoint, scene-context, or capture-protocol bias, all of which can weaken cross-dataset transfer. Future work should incorporate bias-aware evaluation, stronger view/illumination augmentation, occlusion-aware training, and adaptation strategies for low light, blur, partial occlusion, and background clutter.

### 5.3 Ethical considerations and deployment constraints

PHARL prioritizes scientific analysis and representation learning over direct severity prediction. At inference time, only RGB input is required; pose sequences, physics outcomes, and related kinematic metadata are used offline during training and evaluation only. Physics simulation is conducted on anonymized, non-identifiable data. The framework is intended for research and analytical use, not for clinical or safety-critical decision-making.

## 6 Conclusion

We presented PHARL, a physics-regularized representation learning framework for fall-motion analysis without explicit injury annotations. PHARL addresses a central limitation of purely data-driven pipelines: motion-level similarity does not necessarily imply physics-consistent outcomes. By using simulation to construct outcome-level equivalence relations (instead of predicting outcomes directly), PHARL injects physical inductive bias as a structural regularizer for contrastive learning. PHARL offers three practical benefits: (1) it avoids costly injury annotation, (2) it confines simulation to training while preserving efficient feed-forward inference, and (3) it provides a principled mechanism for incorporating domain knowledge through physics-derived structure. Experiments on four public datasets show consistent gains in contact-aware representation quality over motion-only baselines under linear-probe and geometry-based diagnostic protocols. Future work may extend this framework to other contact-rich activities, incorporate environment-specific physical interactions beyond ground contact, and explore multimodal extensions.

## Data Availability

The original contributions presented in this study are included in the article/supplementary material. The publicly available fall datasets used in this work were obtained in full compliance with the respective licensing agreements of each dataset. Further inquiries regarding the study's contributions can be directed to the corresponding author.

## Footnotes

1 We use “outcome” to refer to physics-derived contact configurations (*Supported, Trunk, Head*), not clinical injury severity or severity scores.
